# Development and validation of a test instrument for the assessment of perceived basic motor competencies in first and second graders: the SEMOK-1-2 instrument

**DOI:** 10.3389/fpsyg.2024.1358170

**Published:** 2024-03-27

**Authors:** Kathrin Bretz, Anne Strotmeyer, Harald Seelig, Christian Herrmann

**Affiliations:** ^1^Physical Education Research Group, Zurich University of Teacher Education, Zurich, Switzerland; ^2^Department of Exercise and Health, Paderborn University, Paderborn, Germany; ^3^Department of Sport, Exercise and Health, University of Basel, Basel, Switzerland

**Keywords:** sport, motor development, Physical Education, sport participation, childhood, test development

## Abstract

Both actual motor competencies (AMC) and perceived motor competencies (PMC) play an important role in motor development research and children's physical and psychological development. PMC refer to children's perceptions of their motor competencies. To assess the PMC of first and second grade children (aged 6–9 years), the SEMOK-1-2 instrument was developed. The instrument is aligned to the validated MOBAK-1-2 instrument which assesses AMC in the competence areas “object movement” and “self-movement” Accounting for possible reading difficulties in younger children, the motor tasks and answer options were illustrated and explained verbally. The purpose of this study was to test and validate the SEMOK-1-2 instrument and investigate the associations between the constructs AMC, PMC and physical activity (PA), whereby PA was measured by the participation in team and individual sports. Data from *N* = 404 pupils in the German-speaking part of Switzerland from first and second grades (*M* = 7.8 years, SD = 0.69, 49% boys) were analyzed. Confirmatory factor analyses were conducted to test the factorial validity of the SEMOK-1-2 instrument. Structural equation models were used to investigate the association between the constructs. The analyses confirmed a two-factor structure with the factors PMC “object movement” and PMC “self-movement”, corresponding to the factors existing in the MOBAK-1-2 instrument. Latent correlations between AMC factors and the corresponding PMC factors were *r* = 0.79 for “object movement” and *r* = 0.76 for “self-movement”. Associations with external criteria and covariates, such as sex, were associated with both AMC and PMC. Analyses also revealed that children who participated more often in individual and team sports showed higher levels in both AMC and PMC. The confirmation of the two-factorial structure of the SEMOK-1-2 instrument and the associations between AMC and PMC as well as external criteria indicate construct and criterion validity. The SEMOK-1-2 instrument can be economically utilized for assessing PMC and is also suitable for the monitoring of PMC in the context of Physical Education.

## 1 Introduction

Based on reflexive and rudimentary movements and determined by socio-cultural and geographical influences, children develop and extend their repertoire of motor competencies (e.g., kicking, running; Herrmann, 2018; Hulteen et al., 2018). In childhood, motor competencies are the prerequisites to participate in the culture of sport and movement. Since actual motor competencies (AMC) and perceived motor competencies (PMC) are seen to be driving influencing factors of physical activity (Lopes et al., 2021), the investigation of AMC and PMC as well as their interplay has been the focus of several studies (Barnett et al., 2022; Estevan et al., 2023).

Deficits in AMC have been revealed worldwide. For instance, Bolger et al. (2021) showed, that children in preschool age (3–5 years) show average AMC, while children aged 6–10 years show below-average levels compared to the normative data of the used test instrument. Deficits regarding physical activity could also be observed. The World Health Organization (WHO) recommends at least 60 min of moderate to vigorous physical activity per day for children and adolescents. However, the WHO Global Status Report on physical activity shows that the physical activity recommendations are not achieved by 81% of adolescents (WHO, 2022). Therefore, the assessment and investigation of AMC and PMC as determinants of PA is important, as high levels of both AMC and PMC are positively related to good health attributes (Barnett et al., 2022; Estevan et al., 2023).

The development of AMC is seen as a main goal of Physical Education in school and AMC are considered as important components for sport-specific skills and an active lifestyle over the lifespan (Bildungsdirektion des Kantons Zürich, 2017). Moreover, they are necessary to overcome the proficiency barrier and to develop sport-specific skills (Hulteen et al., 2018). This sport-specific skills can used for participation in different sports and can result in a lifetime of physical activity (Hulteen et al., 2018). All developmental steps are depending on and influenced by biological (e.g., sex) and environmental factors (e.g., participation in learning situations) and associated with physical (e.g., weight status) and psychological (e.g., perceived competence) attributes (Hulteen et al., 2018; Lopes et al., 2021).

AMC refer to the ability to perform various motor tasks, including coordinating gross and fine movements for everyday activities (Robinson et al., 2015; Almeida et al., 2023). As the term “motor competence” is based on several definitions, AMC will be used as an umbrella term to include different definitions and constructs.

Different approaches lead to the use of different test instruments to measure AMC, e.g., Test of Gross Motor Development (TGMD, Webster and Ulrich, 2017), the “Körperkoordinationstest für Kinder” (KTK, Kiphard and Schilling, 2017), or the MOBAK instruments (in German: Motorische Basiskompetenzen, Herrmann, 2018). The Test of Gross Motor Development (TGMD, Webster and Ulrich, 2017) is a process-oriented assessment and examines qualitative aspects of movement (e.g., movement patterns). The test relates to the construct of fundamental movement skills (FMS) which can be measured in the subscales “locomotor skills” and “object control skills.” The KTK (Kiphard and Schilling, 2017) is a product-oriented test which measures quantitative outcomes of motor performance (e.g., number of correct jumps). The KTK instrument includes four items which assess gross body control, coordination, and dynamic balance. The MOBAK instruments assess basic motor competencies and refer to the newly developed approach which theoretically substantiates basic motor competencies as an educational goal in Physical Education (Herrmann et al., 2016). The MOBAK instruments were developed for preschool and primary school (first to sixth grade). Accordingly, the difficulties of the test items refer to the educational goals of the curriculum (Herrmann and Seelig, 2017a; Herrmann, 2018; Herrmann et al., 2020). With the MOBAK instruments, the basic motor competencies can be assessed in the competence areas “object-movement” and “self-movement.”

Children's self-perceptions are based on concrete, observable characteristics. PMC are an important construct in the context of motor development and are also defined as an educational goal in Physical Education (Stodden et al., 2008; Högger, 2015). PMC refer to the perception of the motor competencies a child thinks to have. As children with low PMC will probably engage less in sports and PA than children with higher levels of PMC, it is seen as an important factor in motor development research (Stodden et al., 2008; Almeida et al., 2023). Estevan and Barnett (2018) have integrated perceived motor competence (PMC) into the hierarchical and multidimensional model of self-concept by Shavelson et al. (1976). Further constructs are differentiated within PMC, analogous to AMC, e.g., PMC in “locomotor skills” and PMC in “object control skills,” analog to the construct of “fundamental movement skills” and the dimensions of the TGMD.

Both AMC and PA are associated with improved physical and mental health parameters (Lubans et al., 2010; Robinson et al., 2015; Pate et al., 2019). As mentioned before, national and international studies show, that the recommendations given by the WHO are not achieved by children and adolescents (Hänggi et al., 2022; WHO, 2022). PA is relevant from a health-related perspective throughout the whole lifespan. In early childhood, PA, e.g., running or balancing, is elementary for the development of AMC. Later in childhood, an inverse relationship could be observed, as AMC are relevant for further PA (Stodden et al., 2008). PA can be assessed in different ways. Wearables, such as accelerometers, can be used to measure PA quantitatively. On the other hand, questionnaires can be used to investigate the content of physical activity, such as the participation, type, and frequency in sports clubs. Especially regarding the approach of an idea of participation in the culture of sport and movement, the content, in which children move is relevant (Neuber and Golenia, 2018).

Stodden et al. (2008) postulated a conceptual model and within it a reciprocal and developmentally dynamic relationship between AMC and PA. Children with low levels of AMC cannot overcome the proficiency barrier to develop sport specific motor skills and do not reach the adequate levels of PA and health related fitness. This can result in a higher risk of obesity and a negative spiral of engagement, whereas children with a higher level of AMC can result in a positive spiral of engagement. This relationship can also be mediated by PMC and health related fitness—depending on the phase of childhood. In early childhood, both AMC and PA are influenced by PMC, whereas reciprocal relationships between PMC and AMC as well as PMC and PA were postulated. PMC is seen as an important factor in motor development as children with low PMC will probably engage less in sports and physical activity than children with high PMC (Stodden et al., 2008; Almeida et al., 2023). In addition, Barnett et al. (2022) conducted a systematic review related to the model and found that the evidence on the relationship between AMC and PMC is insufficient due to cross-sectional studies with different aligned instruments. In a longitudinal study by Utesch et al. (2018), the interplay between AMC and its perception was found to be an important aspect for PA in childhood. Children with high levels of AMC did not necessarily have high levels of PMC and vice versa. The authors concluded that an accurate self-perception of AMC is a significant predictor of PA. Estevan and Barnett (2018) suggested to use task-specific and aligned instruments to measure both AMC and PMC to ensure that the constructs of interest are represented in both assessments.

## 2 Assessment of PMC in children

Various instruments have been developed to measure PMC, some of which are directly based on and aligned to measure instruments for AMC. McGrane et al. (2016) developed the physical self-confidence scale to assess adolescents perceived confidence at performing specific skills, of which some questions are based on the skills assessed in the TGMD. Herrmann and Seelig (2017b) developed an instrument to assess the PMC of fifth- and sixth-graders (SEMOK-5-6), whereby the questions were aligned to the MOBAK instrument for the assessment of AMC in fifth and sixth grade students (Herrmann and Seelig, 2017a). Due to the low level of reading literacy at the beginning of primary school, instruments are needed specifically for this target group. In the following section, instruments for the assessment of PMC are presented, whereby a newly developed instrument for the assessment of PMC (SEMOK-1-2; in German: Selbstwahrnehmung motorischer Basiskompetenzen) will be introduced.

### 2.1 Pictorial scales in assessments for children

The assessment of different constructs in young children who cannot read requires specific test instruments, as written questionnaire cannot be used. Attempts to solve this problem led to the development of pictorial instruments or scales. Sauer et al. (2020) conducted a review of pictorial scales in research and practice and developed recommendations for the development of pictorial scales. The review shows, that there is a lack of stringent methodological approaches in the development and validations of these instruments.

There are pictorial instruments which were developed for preschool and primary school children, e.g., to assess children's fears. Muris et al. (2003) used an illustration of a Koala for pictorial response options, representing three different levels of fear. The Koala Fear Questionnaire uses different pictures representing possible fear situations, and the child could choose one of the three Koala faces showing emotional expressions (“no fear,” “some fear,” “a lot of fear”).

In the field of motor competencies, pictorial response options are used in the Pictorial Movement Skill Competence (PMSC) instrument, which alignes to the TGMD (Barnett et al., 2015; Webster and Ulrich, 2017). Due to the young age of the children, the instrument is administered one-on-one to each child and takes about 15–18 min per child. In addition, gender-specific versions for girls and boys are used (Barnett et al., 2015; Estevan et al., 2019). By using this instrument, a child first has to choose between two pictures, whereby one represents the success and the other one the failure of the task. Following this, he/she has to specify whether he/she is “really good” or “pretty good” for the success picture and “sort of good” or “not that good” for the failure picture.

### 2.2 Development of the SEMOK-1-2 instrument (in German: Selbstwahrnehmung motorischer Basiskompetenzen)

To assess PMC, instruments which are aligned to the AMC instruments are used. As there is no aligned PMC instrument to the MOBAK instrument for the first and second grade yet, the SEMOK-1-2 instrument has been developed to measure PMC of the children. Based on the review by Sauer et al. (2020), the given recommendations will be addressed below. Moreover, the following points were considered in the development of the instrument: (1) Economic assessment of PMC: in contrast to other instruments that assess the PMC of the children in a one-to-one situation, the developed instrument should be applicable in a class setting. (2) Enforceability despite poor reading skills: to be feasible in a classroom setting, the instrument should not require written instructions. Therefore, the motor tasks were illustrated. (3) Neutral gender and ethnic representation of the illustrated animal: to avoid gender and ethnic representation, the tasks were performed by an illustrated fox instead of illustrated children.

**Figure 1:**
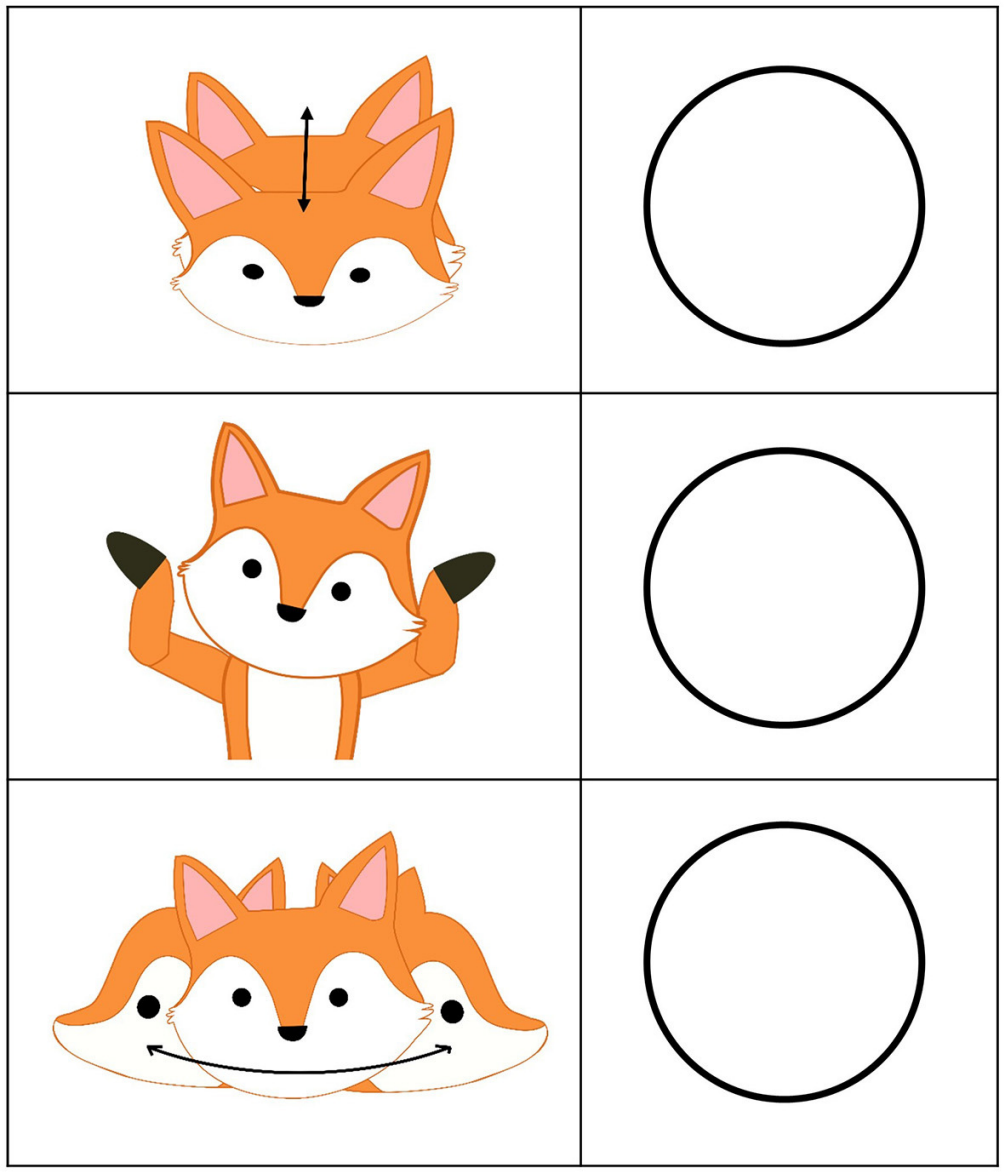
Scale of the SEMOK-1-2 instrument. Pictorial response options: nodding, shrugging shoulders, shaking head (Bretz et al., 2023, p. 5).

Based on the MOBAK-1-2 instrument, which assesses the AMC of the children with each four items in the competence areas “self-movement” and “object movement,” the eight PMC items in the SEMOK instrument refer to children's perception of whether they can perform the basic motor requirements (e.g., throwing, catching, balancing, rolling) formulated on the basis of curricular standards in the MOBAK instrument (e.g., “the child can throw a ball against a target”; Herrmann, 2018). A fox named “Foxy” was illustrated performing the tasks. As the motor tasks of the MOBAK instrument can be represented by a picture (Sauer et al., 2020), each task was illustrated, whereby Foxy was performing the task (Bretz et al., 2023, p. 6f). Comparable to Muris et al. (2003), three pictorial response options were illustrated, representing “negative,” “neutral” or “positive” valances (Figure 1).

**Figure 2:**
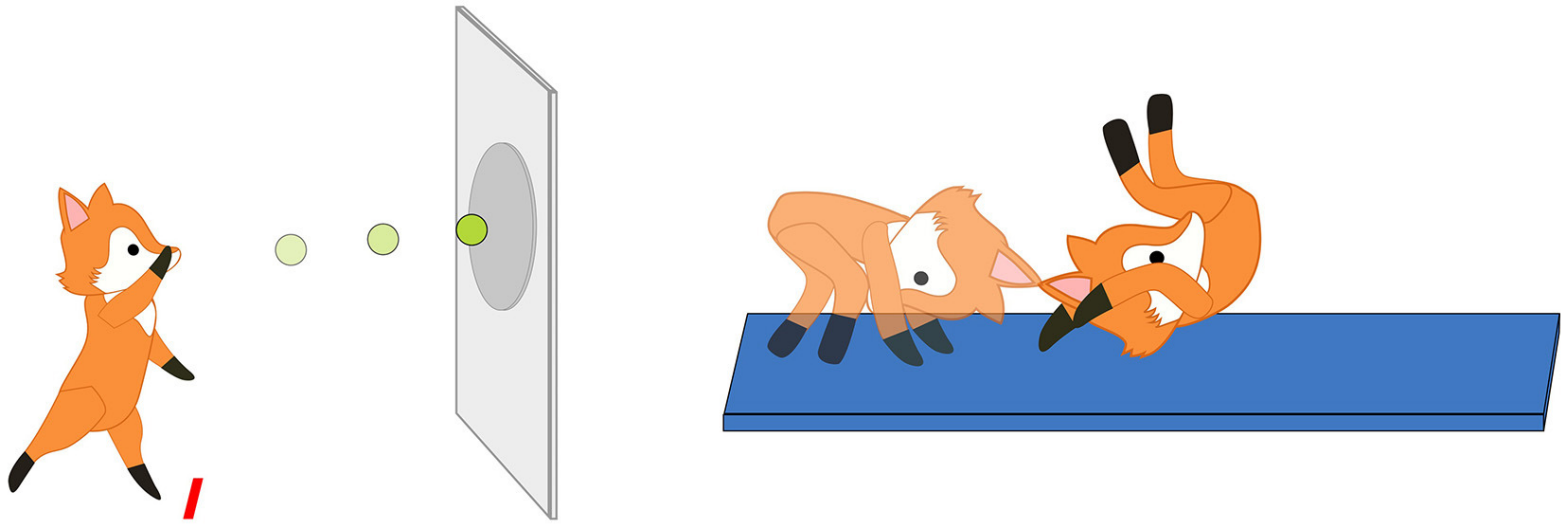
Examples of the illustrated motor tasks “throwing” and “rolling” (Bretz et al., 2023, p. 6, 11).

For the PMC assessment, every child receives a questionnaire with the illustrated test items and the pictorial response options next to each item (Figure 2; Bretz et al., 2023, p. 14f). The questionnaire is presented in paper format, with a front and back page, whereby four tasks displayed one below the other on each page (Bretz et al., 2023, p. 14–15). In the beginning, the pictorial response options (Figure 1) are explained.

The standardized explanation of the pictorial response option is as follows (translated from German, Bretz et al., 2023): “First of all, we will look at the answer options together. In the first picture, Foxy is nodding, which means that Foxy can do the task. In the second picture, Foxy is shrugging its shoulders, which means that Foxy can partially do the task. In the third picture, Foxy is shaking its head, which means that Foxy cannot do the task. Each time there is a circle next to Foxy's head. On your sheet, you will find this box next to each task Foxy is performing. After the task has been explained, you have time to think about how you can do the task and tick one of the circles. Today there is no right or wrong and it is only about yourself. After explaining the pictorial response options, each task is explained separately followed by the sentence “Now think about yourself and tick one of the circles.” After ticking, the children are asked to wait with their arms crossed until all children finish and the next task will be explained. The whole questionnaire takes 10–15 min to complete. The instructions for the items are listed in Table 1. To ensure survey standardization, the test administrators undergo training and are provided with a manual containing the verbal instructions. The motor tasks are not shown but explained verbally only.

**Table 1:** Explanation of the SEMOK-1-2 items (translated from German, Bretz et al., 2023).

**Item**	**Explanation**
1. PMC throwing	Foxy throws a small ball, about the size of a tennis ball, at a target and hits the target.
2. PMC catching	Foxy catches a small ball, about the size of a tennis ball, with both hands. The ball may only be caught with the hands and must not touch the body.
3. PMC bouncing	Foxy bounces a ball on the floor while walking through a narrow passage. The ball can be bounced with one hand or both hands and must not be lost.
4. PMC dribbling	Foxy dribbles a ball with the feet through a narrow passage. The ball must not be lost in the process.
5. PMC balancing	Here we have built a small seesaw. Foxy balances forward over an inverted long bench that tips over halfway. Then Foxy balances backwards and the bench tips back again. Foxy keeps the balance and does not fall off the bench.
6. PMC rolling	Foxy does a somersault. The chin is close to the chest and the back is round. Then Foxy stands up again.
7. PMC jumping	Foxy hops through a parkour of carpet tiles. Foxy hops between the tiles on one leg, straddling the tiles with both legs.
8. PMC running	Foxy runs sideways from one cone to the other and then back again.

### 2.3 Aim of the study

The MOBAK instruments for the assessment of AMC (Herrmann and Seelig, 2017a; Herrmann, 2018), have been used in numerous studies and are a widely accepted instrument for assessing AMC (Strotmeyer et al., 2020; Herrmann et al., 2021; Wälti et al., 2022; Carcamo-Oyarzun et al., 2023). To assess PMC, aligned instruments for the fifth and sixth grade were developed (Herrmann and Seelig, 2017b) and adapted for the third and fourth grade (Strotmeyer et al., 2022). With the development of the SEMOK-1-2 instrument, the assessment of PMC in younger children can also be assessed.

Against this background, the study aims first to test the construct of the developed SEMOK-1-2 instrument to examine the assumed two-factorial structure, analogous to the MOBAK instrument with the factors “object movement” and “self-movement.” Second, to investigate the criterion validity by relating the AMC and PMC constructs. Third, to examine associations between the AMC and PMC constructs and covariates (age, sex and BMI) as well as sports club participation.

## 3 Materials and methods

The present validation study was a cross-sectional study based on the second measurement point of the longitudinal study “Development of basic motor competencies in children (EMOKK)” (2021–2025), funded by the Swiss National Science Foundation (SNSF; Grant number 200840).

### 3.1 Actual motor competencies

To measure AMC, the MOBAK instrument for the first and second grade of primary school was used (MOBAK-1-2, Herrmann, 2018). With the MOBAK instrument, AMC can be measured in the competency area “object movement” and “self-movement,” operationalized with four items per competency area (object movement: throwing, catching, bouncing, dribbling; self-movement: balancing, rolling, jumping, running) (Herrmann, 2018). Each test item describes a standardized task with corresponding assessment criteria. During the test, each child had two attempts to try to achieve the motor task (no trial run). The two single attempts were rated on a dichotomous scale (0 = failed, 1 = successful), and the individual results were summed up to form the final item score (0 points = no successful attempts, 1 point = one successful attempt, 2 points = two successful attempts). The scores for the test items throwing and catching were calculated differently. In these cases, the children had six attempts each, and the number of successful attempts was recorded. Subsequently, 0–2 successful attempts were scored as 0 points, 3–4 successful attempts as 1 point, and 5–6 successful attempts as 2 points. For each competency domain, a maximum of eight points could be achieved (for details, see Herrmann, 2018). Data was collected in class during a regular 45-min Physical Education lesson. The class was divided into small groups of three to four children each and led through the eight test stations by trained testers. The testers provided a standardized explanation and one demonstration of each test item. The factorial validity of the MOBAK instrument for primary school (MOBAK-1-2) has already been investigated and confirmed in various studies (Herrmann et al., 2016, 2019a). The weight and height of the children was measured as part of the MOBAK test to calculate the Body-Mass-Index (BMI).

### 3.2 Parent questionnaire

The parents of the children completed a questionnaire. In addition to general information about the child, the questionnaire contained questions about the sports activity of the child, e.g., how often the child plays outside or participates in organized sports activities (in detail, see Herrmann et al., 2023). Parents were asked whether their children participate in a sports club and, if so, to what extent (frequency per week) and in which sports (up to three answers possible, either by ticking predefined sports or as open answers). The type and frequency data were assigned to the categories team sports (e.g., football, handball) or individual sports (e.g., swimming, gymnastics) and summed up. This resulted in sum values for the variables frequency of team sports and frequency of individual sports.

### 3.3 Perceived motor competencies

PMC were assessed before measuring the AMC. Therefore, the children filled out the questionnaire during the last 15 min of the regular lesson in their classroom before the Physical Education lesson or on another day before the AMC assessment. The procedure and instructions were briefly described in the previous section.

For subsequent analyses, the answers given to the pictorial response options were coded: “positive”/nodding = 2 points, “neutral”/shrugging shoulders = 1 point, “negative”/Shaking head = 0 points. Following, the points per competency domain were summed up (0–2 points per item, eight points per competency domain). This means that the tasks in the PMC instruments refer to the tasks in the AMC instrument but also that the scores of the AMC and PMC instruments are aligned.

### 3.4 Sample

AMC and PMC data were collected in the Swiss cantons Basel-Landschaft and Zurich in spring/summer 2023. In total, we contacted parents or legal guardians of 558 children from the first and second grade. Of these children, 404 parents (72.4%), gave their written consent for their children to participate in the study. We included *N* = 404 children (*M* = 7.8 years, SD = 0.69, 49% boys) from 29 classes in the study, with an average class size of *n* = 14. The data was obtained from three different sources (AMC, MOBAK instrument; PMC, SEMOK instrument; PA, parent questionnaire) and was merged. The assessment of AMC, which took place during a Physical Education lesson, involved *n* = 378 children, and data on PMC was collected prior to the assessment in the regular classroom from *n* = 391 children. The parents of *n* = 376 children completed the parent questionnaire at home. The study was conducted in the accordance with the Declaration of Helsinki and approved by the Ethics Committee of the University of Zurich (Nr. 21.2.5, 19.12.2022). Informed consent was obtained from all parents of the participants in this study and the participation was voluntary and could be canceled at any time.

### 3.5 Data analysis

The data processing, descriptive and correlational analyses were conducted with SPSS 28 (IBM Corp., 2021). Multivariate analyses were performed by using Mplus 8.8 (Muthén and Muthén, 2017).

At a manifest level, descriptive statistics were calculated. Therefore, sum values regarding the AMC and PMC single items were calculated for AMC “object movement,” AMC “self-movement,” PMC “object movement” and PMC “self-movement.” The mean values were calculated for the total sample and separately for girls and boys. We calculated 95% confidence intervals and Cohen's *d*. As effect sizes Cohen's d were interpreted as small (*d* = 0.10), medium (*d* = 0.50) and large (*d* = 0.80) (Cohen, 1988). Regarding PA, the mean values of frequency in team and individual sports were also calculated for the total sample and girls and boys separately. Moreover, we calculated Spearman correlations for non-parametric data to investigate the associations between the constructs on a manifest level.

Modeling latent structures was carried out in three steps. First, the factorial validity of the SEMOK instrument, which measures PMC, was examined by calculating confirmatory factor analyses (CFA). Second, the criterion validity was investigated, whereby the AMC and the PMC factors were related. Third, correlations with covariates (age, sex, BMI) and the frequency of sports club participation were examined. Influences of the multilevel structure (students from different classes) were tested with the help of interclass correlations (ICC).

#### 3.5.1 Missing data handling

There were missing values due to the different data sources (AMC assessment, PMC assessment, parent questionnaire) and partly different survey days. Some children participated in the AMC assessment, but not in the PMC assessment and vice versa. Moreover, not all parents filled out the parent questionnaire. From children who participated in the assessment of AMC, frequencies of missing values ranged from 0.5% (AMC jumping) to 4.2% (AMC balancing). In the PMC assessment, missing values were only identified for PMC bouncing and PMC dribbling (both 0.5%). Regarding the parent questionnaire, 5.1% of the parents who filled out the questionnaire did not answer the question about sports club participation. Missing values were estimated via the full information maximum likelihood (FIML) algorithm. The FIML procedure is a conservative and well-established procedure in educational research. The FIML procedure prevents bias in the sample composition by preventing a reduction in the sample size (Urban and Mayerl, 2014).

#### 3.5.2 Modeling latent structures

Construct validity of the SEMOK-1-2 instrument was investigated by calculating CFAs.

*Model 1a:* Due to the two-factorial structure of the MOBAK instrument (Herrmann et al., 2015) as well as the previous SEMOK-5-6 instruments (Herrmann and Seelig, 2017b; Strotmeyer et al., 2022), it was assumed that the developed SEMOK-1-2 instrument would also have a two-factorial structure. Therefore, the factor structure of the SEMOK instrument was tested by calculating a two-factorial CFA with the factors “PMC object movement” (PMC throwing, PMC catching, PMC bouncing, PMC dribbling) and “PMC self-movement” (PMC balancing, PMC rolling, PMC jumping, PMC running).

*Model 1b:* Based on model 1a, the covariates sex, age and BMI were included as covariates in the model. Modification indices (MI) can be used to check which relaxation of restrictions leads to a statistically significant improvement of the model (Geiser, 2011). In this model, we requested the modification indices (MI = all) for the direct effect of the covariates.

Criterion validity of the SEMOK-1-2 instrument was investigated by calculating associations between AMC and PMC of the children.

*Model 2:* In model 2, the relationship between the AMC and PMC factors was investigated to test concurrent validity. Therefore, we calculated a confirmatory factor analysis with the four factors AMC “object movement,” AMC “self-movement,” PMC “object-movement” and PMC “self-movement.” Sex, age and BMI were integrated in the model as covariates.

Finally, the associations between AMC, PMC and PA were calculated.

*Model 3*: In Model 3 we investigated associations between AMC, PMC, and PA. Next to the latent AMC and PMC factors, the manifest factors of frequency of team sports and frequency of individual sports were included. Age, sex, and BMI were included as covariates in the model.

In all models, we treated the AMC and PMC as ordinal-scaled data. Accordingly, we used the mean- and variance-adjusted weighted least squares (WLSMV) estimator. We accounted for dependencies within the multilevel structure (0.01 ≤ ICC ≤ 0.19; Table 2) in all models by correcting the standard error with the “type = complex” function for nested datasets implemented in Mplus. The goodness of fit of the models was assessed using fit indices proposed in the literature (Schreiber et al., 2006). Effect sizes were interpreted as small (*r* > 0.10, β > 0.05), medium (*r* > 0.30, β > 0.25), or large (*r* > 0.50, β > 0.45) (Cohen, 1988; Peterson and Brown, 2005).

**Table 2:** Descriptive values and interclass-correlations (ICC) of the actual (AMC) and perceived (PMC) motor competency domains and the frequencies of team and individual sports.

	**Overall**			**Boys**		**Girls**		***d***
	***N***	***M*** **CI 95%**	**ICC**	***n***	***M*** **CI 95%**	***n***	***M*** **CI 95%**
AMC object movement^a^	369	5.76 (5.60; 5.93)	0.11	179	6.23 (6.01; 6.45)	190	5.33 (5.09; 5.57)	0.57
AMCsSelf-movement^a^	352	5.70 (5.52; 5.89)	0.19	174	5.46 (5.18; 5.74)	178	5.94 (5.69; 6.19)	−0.27
PMC object movement^a^	383	6.33 (6.18; 6.48)	0.03	190	6.97 (6.80; 7.14)	193	5.70 (5.50; 5.90)	0.97
PMC self-movement^a^	383	7.23 (7.14; 7.33)	0.01	190	7.17 (7.02; 7.33)	193	7.30 (7.17; 7.42)	−0.12
Frequency team sports^b^	369	0.54 (0.45; 0.64)	0.04	182	0.84 (0.67; 1.00)	187	0.26 (0.17; 0.35)	0.64
Frequency individual sports^b^	369	1.08 (0.96; 1.19)	0.05	182	0.89 (0.75; 1.03)	187	1.25 (1.08; 1.42)	−0.34

a Range: 0–8.

b Days per week.

## 4 Results

Table 2 shows the descriptive values of AMC and PMC as well as the frequency of sport participation in team and/or individual sports. Boys showed better AMC in “object movement” (*d* = 0.57) than girls while girls had better AMC in “self-movement” (*d* = −0.27). Regarding PMC, boys rated themselves higher than girls in PMC “object movement” (*d* = 0.97). Most of the children whose parents filled out the questionnaire, were a member of a sports club (83.8%). Of the children, who participated in a sports club, 59.1% were active only in individual sports, 16.8% were participating only in team sports and 24.1% of the children were participating in both individual and team sports. Regarding the participation in organized sports, girls engaged more in individual sports (*d* = −0.34) than boys whereas boys engaged more in team sports (*d* = 0.64).

### 4.1 Factorial validity of the SEMOK-1-2 instrument

**Figure 3:**
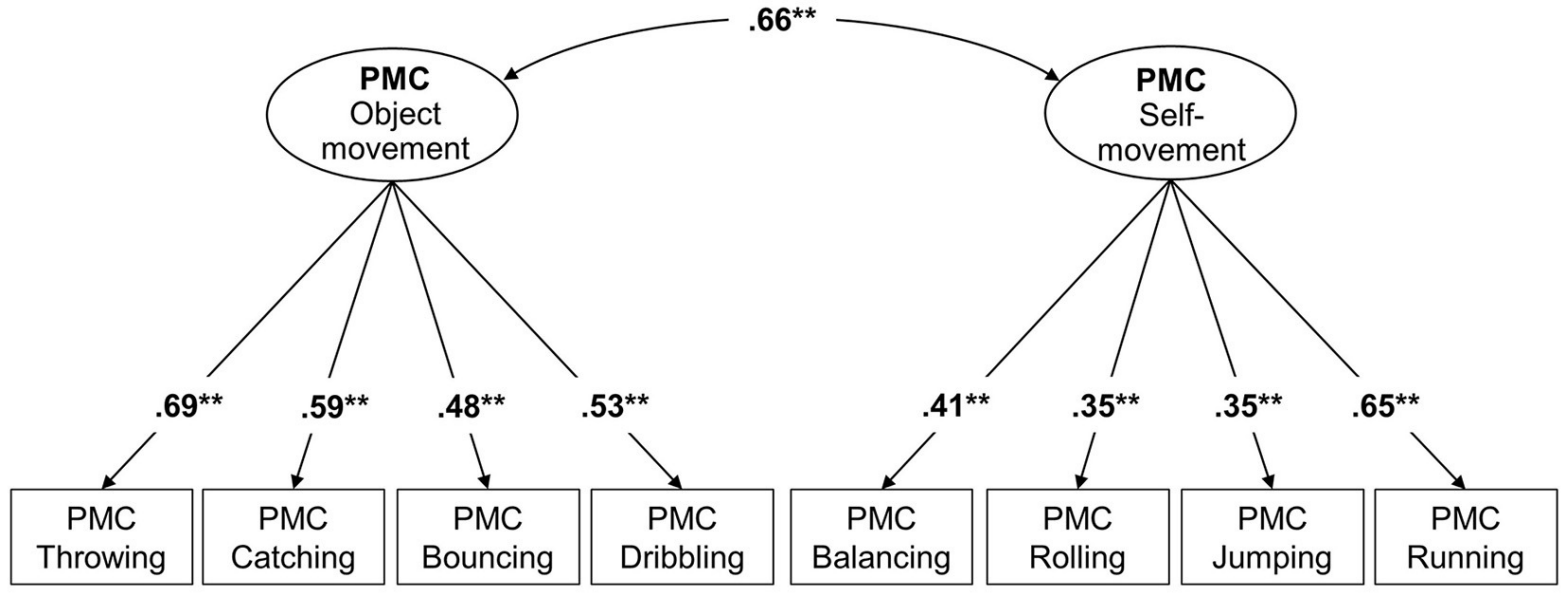
Two-factorial confirmatory factor analysis for the SEMOK instrument with the factors PMC “object movement” and PMC “self-movement” (Model 1a). **p < 0.01.

Model 1a: The CFA with the two factors PMC “object movement” and PMC “self-movement” showed a good model fit (χ^2^ = 26.447; df = 19; *p* = 0.118; CFI = 0.940; RMSEA = 0.032; *N* = 391). The factor loadings ranged from β = 0.35 to β = 0.69 (Figure 3). The correlation between the factors PMC “object movement” and PMC “self-movement” was *r* = 0.66 (*p* < 0.001).

Model 1b: Based on model 1a, the covariates sex, age and BMI were integrated to model 1b. The model showed a good model fit, whereby the model fit increased slightly in comparison to the model without the covariates. (χ^2^ = 45.14; df = 37; *p* = 0.017; CFI = 0.969; RMSEA = 0.023; *N* = 377). No modifications concerning the minimal value (MI > all) were suggested. Sex was found to have a significant effect on the factor PMC “object movement” (*r* = −0.63, *p* < 0.001), but not on the factor PMC “self-movement” (*r* = 0.06, *p* = 0.457). Age and BMI did not show significant effects on PMC factors. The correlation between PMC “object movement” and PMC “self-movement” was *r* = 0.93 (*p* < 0.001).

Both models and the resulting model fits showed that the assumed two-factor structure with the factors PMC “object movement” and PMC “self-movement” could be confirmed.

### 4.2 Criterion validity of the SEMOK-1-2 instrument

**Figure 4:**
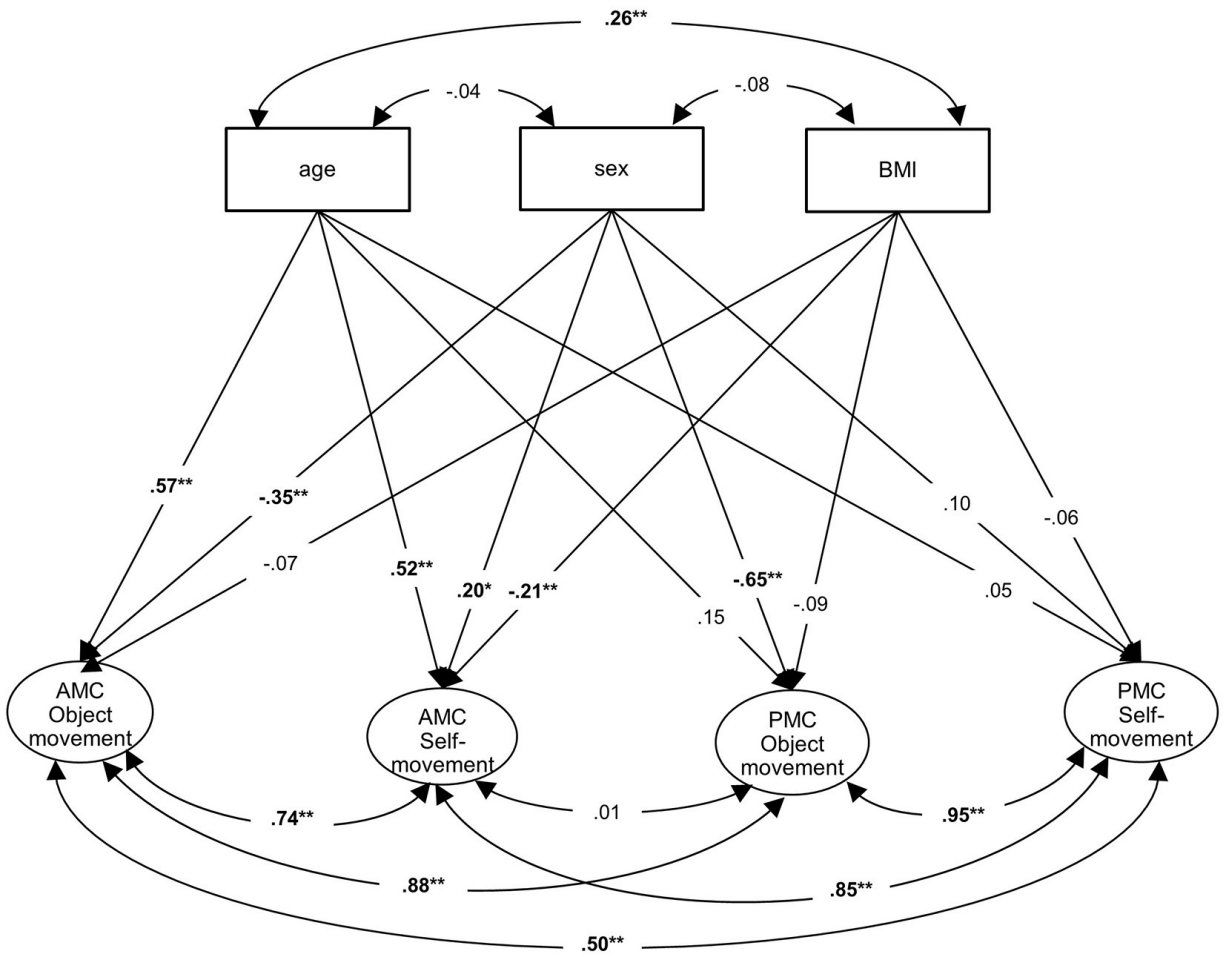
Structural equation model (SEM) with the factors AMC “object movement,” AMC “self-movement,” PMC “object movement” and PMC “self-movement” with the covariates age, sex and BMI (Model 2). *p < 0.05, **p < 0.01.

To investigate the criterion validity, the associations between the AMC and PMC factors were calculated. The four-factor confirmatory analysis with the factors AMC “object movement,” AMC “self-movement,” PMC “object movement” and PMC “self-movement” with the covariates resulted in a good model fit (χ^2^ = 173.651; df = 134; *p* = 0.012; CFI = 0.909; RMSEA = 0.027; *N* = 404). The correlation between the factors AMC “object movement” and PMC “object movement” was *r* = 0.88 (*p* < 0.001) and between the factors AMC “self-movement” and PMC “self-movement” *r* = 0.85 (*p* < 0.001). There was no correlation between PMC “object movement” and AMC “self-movement” (*r* = 0.01, *p* = 0.953) but a significant correlation between PMC “self-movement” and AMC “object movement” (*r* = 0.50, *p* = 0.011; Figure 4). The correlation between AMC “object movement” and AMC “self-movement” was *r* = 0.74 (*p* < 0.001) and between PMC “object movement” and “self-movement” *r* = 0.95 (*p* < 0.001).

### 4.3 Associations between AMC, PMC and PA

Model 3: Based on model 2, frequency of team sports and frequency of individual sports, were included as manifest variables in model 3 to investigate the associations between AMC, PMC and PA. Moreover, sex, age and BMI were included as covariates.

The model showed a good model fit (χ^2^ = 200.149; df = 158; *p* = 0.013; CFI = 0.914; RMSEA = 0.026; *N* = 404). The correlations are shown in Table 3 (below the diagonal).

**Table 3:** Correlations between the AMC and PMC factors, frequency of team and individual sports and sex, age and BMI as covariates (Model 3).

	**First-order correlations**						**Zero-order correlations**		
	**(1)**	**(2)**	**(3)**	**(4)**	**(5)**	**(6)**	**(7)**	**(8)**	**(9)**
(1) AMC object movement		**0.27**	**0.28**	0.05	**0.12**	0.02	**−0.29**	**0.40**	0.08
(2) AMC self-movement	**0.75**		0.10	**0.18**	0.09	0.04	**0.16**	**0.32**	−0.02
(3) PMC object movement	**0.89**	<0.01		**0.22**	**0.22**	−0.02	**−0.45**	**0.13**	0.03
(4) PMC self-movement	**0.50**	**0.85**	**0.94**		0.04	**0.11**	−0.01	0.09	−0.02
(5) Frequency team sport	**0.20**	0.12	**0.53**	**0.21**		**−0.19**	**−0.35**	0.05	0.08
(6) Frequency individual sport	0.05	0.11	−0.05	**0.39**	**−0.21**		**0.18**	0.07	0.06
(7) Sex	**−0.36**	**0.20**	**−0.65**	0.10	**−0.30**	**0.17**		−0.05	−0.10
(8) Age	**0.56**	**0.52**	0.14	0.05	0.04	0.05	−0.04		**0.22**
(9) BMI	−0.08	**−0.21**	−0.09	−0.06	0.06	0.02	−0.08	**0.26**	

Correlations in bold are significant (p < 0.05). Latent correlations below the diagonal, manifest correlations above the diagonal; sex: 0 = boys, 1 = girls.

Older children performed better in AMC “object movement” (*r* = 0.56, *p* < 0.001) and AMC “self-movement” (*r* = 0.52, *p* < 0.001) than younger children. Strong correlations were also found between sex and AMC and PMC as well as sport participation. Boys performed better in AMC “object movement” (*r* = −0.36, *p* < 0.001) than girls, whereas girls were better in AMC “self-movement” (*r* = 20, *p* = 0.003). Regarding PMC, boys rated themselves higher in “object movement” (*r* = −0.65, *p* < 0.001). Correlations between PA and sex were also found. Boys participated more often in team sports (*r* = −0.30, *p* < 0.001), whereas girls were more active in individual sports (*r* = 0.17, *p* < 0.001). The frequency of team and individual sports was also associated with PMC. Moreover, there were significant correlations between the frequency of team sport and AMC “object movement” (*r* = 0.20, *p* < 0.001), PMC “object movement” (*r* = 0.53, *p* < 0.001) and PMC “self-movement” (*r* = 0.21, *p* = 0.037). Associations with frequency of individual sports were found with PMC “self-movement” (*r* = 0.39, *p* < 0.001).

The partial Spearman correlations (Table 3, above the diagonal) also showed correlations between AMC and PMC in “object movement” (*r* = 0.28, *p* < 0.001) and “self-movement” (*r* = 0.18, *p* < 0.001). Moreover, correlations between PMC “object movement” and the frequency in team sport (*r* = 0.22, *p* < 0.001) as well as PMC “self-movement” and the frequency in individual sport (*r* = 0.11, *p* = 0.048) were found. The same correlations were found at the latent and manifest levels, although they were lower at the manifest level, as expected.

## 5 Discussion

PMC is seen as an important factor in the context of motor development. An instrument was developed to measure PMC in children in first and second grade, as there was no instrument to measure PMC aligned to the MOBAK-1-2 instrument. Because of the young age of the children and the poor reading skills, illustrated tasks were developed, supported by verbal instructions. The aim of this study was to test construct and criterion validity of the newly developed SEMOK-1-2 instrument and to investigate the associations between AMC, PMC and PA. In the following, the investigated construct and criterion validity are discussed.

Regarding the factorial validity the two-factorial structure with the two factors PMC “object movement” and PMC “self-movement” was confirmed, equivalent to the two-factor structure of the MOBAK instruments. Due to the high correlation between the PMC factors “object movement” and “self-movement” (*r* = 0.95, *p* < 0.001), a one-factor model was also tested, but this resulted in a poorer model fit than the two-factor solution. It is therefore assumed that the two-factor model is the better solution. Thus, it can be seen, that both the MOBAK and SEMOK instruments consistently show this two-factor structure with the factors “object movement” and “self-movement” (Herrmann et al., 2015; Herrmann and Seelig, 2017a,b,c; Strotmeyer et al., 2022).

Integrating sex, age and BMI as a covariate resulted in better model fits. Regarding the modification indices, no relaxation of the restrictions would lead to an improvement of the model and can be taken as an indication that there was no difference in the model regarding sex, age and BMI.

In terms of criterion validity, strong positive correlations between children's AMC and PMC were found, especially at the latent level (Model 2). These correlations indicate that the children's assessed PMC are related to the relevant criterion of children's AMC. The correlation between AMC and PMC was higher than in the study by Strotmeyer et al. (2022) (“object movement”: *r* = 0.45, *p* < 0.01; “self-movement”: *r* = 37, *p* < 0.01) and similar to the study by Herrmann and Seelig (2017b) (“object movement: *r* = 0.70, *p* < 0.001; “self-movement”: *r* = 0.76, *p* < 0.001). The high correlation between the constructs could possibly be due to the high alignment between the AMC and PMC instruments. Other studies show low to moderate correlations between AMC and PMC in children (De Meester et al., 2020). However, it is possible that not only the alignment between the instruments but also the alignment between the scales is decisive for the strength of the correlation.

Differences between girls and boys appeared in model 1c, with boys rating themselves better than girls. That PMC in “object movement” was higher in boys than in girls, is also in line with the literature (De Meester et al., 2016; Herrmann and Seelig, 2017b; Niemistö et al., 2019; Martínez-González et al., 2022). In addition to PMC, differences in AMC were also found between boys and girls. Boys performed better in “object movement,” whereas girls performed better in “self-movement.” The result that boys are better in object movement and girls are better in self-movement has also been found in other studies with children from different age groups (Herrmann et al., 2019b; Wälti et al., 2022). Differences between boys and girls were also found regarding their sport participation. Boys participated more often in team sports (e.g., soccer), whereas girls participated in individual sports (e.g., gymnastics). The finding that boys prefer ball games while girls prefer sports such as dancing or gymnastics was also observed in other Swiss and international studies (Gramespacher et al., 2020; Peral-Suárez et al., 2020; Lamprecht et al., 2021).

The high correlation between sex and PMC “object movement” may be due to a link via the participation in sports club. Children, who participated in team sports showed a higher level in both PMC “object movement” (*r* = 0.52, *p* < 0.001) and PMC “self-movement” (*r* = 0.21, *p* = 0.04). Children who took part in individual sports showed higher levels only in PMC “self-movement” (*r* = 0.39, *p* < 0.001). A positive association between the organized sport activities and perceptions of “object movement” was also found by Niemistö et al. (2019). As boys participate more often in team sports and ball sports, they enhance their AMC in “object movement.” Gramespacher et al. (2020) found that the differences between boys and girls in their AMC were mediated by club sport participation. Indirect effects of sex on “self-movement” were found through the frequency of individual sports and the frequency of team sports. An indirect effect on “object movement” was found via the frequency of team sports (Gramespacher et al., 2020). This would also be conceivable for PMC. It is also possible, that children who have a higher level of AMC and PMC, tend to participate more often in club sports than children with lower levels of AMC and PMC. As PMC in “object movement” is associated with physical activity over time (Barnett et al., 2008), PMC in “object movement” should be promoted, especially in girls.

There are also some limitations in this study. As the children were interviewed in class, the possibility of mutual influence on the answers to the questions cannot be excluded. Although the test leader pointed out that the questions should be answered independently, a few children communicated their answers to the class. Another limitation is that the illustration shows the motor task in a simplified way. The operationalization into an illustration and a short instruction did not explain all the criteria for passing or failing the motor task. Regarding the item difficulty, ceiling effects could be observed, as the mean values in PMC were high, especially in PMC “self-movement.” This could be due to the three-point-scale, as Estevan et al. (2019) mentioned this limitation also regarding the four-point-scale in the PMSC instrument. The high correlation between PMC “Object movement” und PMC “self-movement” (*r* = 0.95, *p* < 0.001) could also be due to these ceiling effects. It should be also considered that young children tend to overestimate their own abilities (Harter, 1999).

What should be considered in future studies is, that due to the study design, no retest for reliability analysis could be conducted. Because of the cross-sectional design of the study, no causal interpretations can be made. However, there is little evidence regarding the direction for preschool and primary school age children due to a lack of longitudinal studies (Dreiskämper et al., 2020). This might be due to the resources and time-consuming assessment of PMC in young children. With the SEMOK-1-2 instrument, the PMC of children can be assessed in a more efficient and economical way what might be an advantage for assessments in large samples.

Overall, it was found that the instrument is suitable for assessing PMC in first and second graders. The strength of the instrument is the economic assessment of PMC in a classroom setting facilitated by the illustrated motor tasks and the neutral gender and ethnic representation of the illustrations. Due to the illustration and the organization of questioning the children in their normal class setting the instrument can be used economically in a larger sample. Physical Education teachers could also use the instrument in class to identify children with low PMC and consequently encourage and support them to reflect on their PMC. These results are important for the diagnosis and identification of PMC to promote AMC and thus an active lifestyle.

## Data availability statement

The raw data supporting the conclusions of this article will be made available by the authors, without undue reservation.

## Ethics statement

The studies involving humans were approved by Ethics Committee of the University of Zurich. The studies were conducted in accordance with the local legislation and institutional requirements. Written informed consent for participation in this study was provided by the participants' legal guardians/next of kin.

## Author contributions

KB: Data curation, Formal analysis, Investigation, Methodology, Writing – original draft, Conceptualization. AS: Writing – review & editing. HS: Data curation, Writing – review & editing. CH: Conceptualization, Funding acquisition, Methodology, Project administration, Supervision, Writing – review & editing.
